# Clinical outcomes following intradiscal injections of higher-concentration platelet-rich plasma in patients with chronic lumbar discogenic pain

**DOI:** 10.1007/s00264-022-05389-y

**Published:** 2022-03-28

**Authors:** Cole Lutz, Jennifer Cheng, Meredith Prysak, Tyler Zukofsky, Rachel Rothman, Gregory Lutz

**Affiliations:** 1 Regenerative SportsCare Institute, New York, NY USA; 2 Department of Physiatry, Hospital for Special Surgery, 535 E. 70th Street, New York, NY 10021 USA; 3 Orthobond Corporation, Princeton, NJ USA

**Keywords:** Platelet-rich plasma, Lumbar discogenic pain, Outcomes, Platelets, Historical

## Abstract

**Purpose:**

This study aimed to assess clinical outcomes following intradiscal injections of higher-concentration (> 10 ×) platelet-rich plasma (PRP) in patients with chronic lumbar discogenic pain and to compare outcomes with a historical cohort.

**Methods:**

This retrospective study included 37 patients who received intradiscal injections of higher-concentration (> 10 ×) PRP and had post-procedure outcomes data (visual numerical scale pain score, Functional Rating Index [FRI], and NASS Patient Satisfaction Index). Outcomes were compared to a historical cohort of 29 patients who received intradiscal injections of < 5X PRP.

**Results:**

Pain and FRI scores significantly improved by 3.4 ± 2.5 and 46.4 ± 27.6, respectively, at 18.3 ± 13.3 months following intradiscal injections of > 10 × PRP (p < 0.001). These improvements were greater than those reported by the historical cohort (1.7 ± 1.6 and 33.7 ± 12.3; p = 0.004 and 0.016, respectively). Additionally, the satisfaction rate was higher in patients receiving > 10 × PRP compared to those receiving < 5 × PRP (81% vs. 55%; p = 0.032).

**Conclusions:**

Findings from this study suggest that clinical outcomes can be optimized by using PRP preparations that contain a higher concentration of platelets. Further research is needed to continue to optimize the composition of PRP used to treat patients with lumbar disc disease.

## Introduction

Low back pain (LBP) is the leading cause of disability worldwide [1], affecting an estimated 577 million people at any time [2]. The global burden of LBP is considerable. In the USA alone, the economic burden of LBP is estimated to exceed $250 billion annually in lost wages and treatment costs [3].

The pathophysiology of LBP—while multifactorial—is often associated with damage to the intervertebral disc (IVD). Up to 42% of chronic LBP cases are discogenic in nature [4–6]. The injured IVD has limited self-healing capacity, due to its poor vascular supply, hypoxic microenvironment, and low cell content. Lack of access to blood-based growth factors, along with inflammation of the richly innervated disc, is theorized to play a role in the prolonged pain that is experienced by many patients with chronic lumbar discogenic pain (CLDP) [7].

Improved understanding of the pathophysiological basis of CLDP has led to the ongoing development of targeted intradiscal biologic therapies that aim to facilitate healing by delivering autologous growth factors directly to the site of injury. In particular, platelet-rich plasma (PRP) can be beneficial in regions in which vascularity is minimal, such as the IVD [8, 9], due to various factors that are emitted from the platelets and cells [10]. Several studies have shown short-term or long-term improvements in patient-reported pain and function following intradiscal injections of PRP [8, 11–14].

Although data supporting the use of intradiscal PRP therapy for a subset of patients with CLDP is promising, certain questions remain. Determining the optimal PRP preparation is one such area of clinical interest. Jain et al. demonstrated a positive correlation between higher platelet concentrations and improvements in outcomes in patients receiving intradiscal PRP therapy for discogenic LBP [11]. The majority of studies in the literature have utilized PRP preparations with a -three to fivefold increase in platelet concentrations [8, 13]. Newer preparations have yielded higher concentrations of platelets in PRP. We have previously analyzed the cellular content of PRP that was processed from 118 patients using an Emcyte PurePRP II kit. Greater than tenfold (> 10 ×) increases in platelet concentration were observed in the PRP that was injected in the majority of patients [15]. No study has evaluated patient-reported outcomes following intradiscal injections of PRP with higher platelet concentrations.

The objectives of this study were to (1) determine whether intradiscal injections of PRP with higher platelet concentrations would improve clinical outcomes in patients with CLDP, and (2) compare clinical outcomes to historical data from a previous study utilizing intradiscal injections of PRP with lower platelet concentrations. It was hypothesized that intradiscal injections of PRP with higher platelet concentrations would significantly improve pain and functional outcomes in patients with CLDP, and that these improvements would be greater than those observed in a historical cohort of patients receiving intradiscal injections of PRP with lower platelet concentrations.

## Materials and methods

### Ethics and patient selection

This retrospective cohort study was approved by the Institutional Review Board. Written informed consent was waived, as all data were retrospectively collected from patients’ charts. Patients with CLDP who received intradiscal PRP injections between January 2017 and January 2019 were retrospectively assessed for eligibility at a single outpatient interventional orthopaedic clinic. The inclusion criteria were: (1) chronic refractory low back pain greater than leg pain for > three months; (2) failure of conservative treatments; (3) maintained disc height of at least 50%; (4) lumbar protrusion < 4 mm; (5) higher concentration (> 10 ×) intradiscal PRP procedure; (6) absence of any contraindications (e.g., progressive neurologic deficits, severe spinal stenosis); and (7) available post-injection outcome data. The exclusion criteria were: (1) previous spinal fusion surgery; (2) spondylolysis; (3) disc extrusion or sequestered fragment; (4) severe disc degeneration; (5) missing contact information preventing follow-up; and (6) intradiscal PRP combined with another orthobiologic agent. Patients who had degenerative grade I spondylolisthesis or a previous history of microdiscectomy were permitted to be included in the study.

### Procedure details

PRP was prepared as previously described [15]. Briefly, for each level injected, 60 ml peripheral blood was drawn and processed down to 4 ml PRP. This yielded platelet counts that were increased by at least 10 × , as previously shown [15]. A small 3-ml syringe was used to slowly inject the PRP (2 ml per disc) at low pressures over one to two minutes. Patients received either leukocyte-rich PRP or leukocyte-poor PRP.

### Data collection

Demographic information, duration of pain, and patient-reported outcomes were collected. Baseline patient-reported outcomes included the numerical rating scale (NRS) pain score and Functional Rating Index (FRI). Follow-up patient-reported outcomes included the NRS pain score, FRI, and the North American Spine Society (NASS) Patient Satisfaction Index. The minimum clinically important differences (MCIDs) for NRS pain and the FRI are 2 points and 9 points, respectively [16, 17]. Patient satisfaction was defined as a response of “The procedure met my expectations” or “I improved less than I hoped, but I would undergo the same procedure again for the same results.” Follow-up time points ranged from three to 43 months.

### Statistical analysis

For the descriptive analysis, continuous data are reported as means, standard deviations, and/or ranges, and discrete data are reported as counts and percentages. Patient-reported outcomes data from the 37 patients in the current study were subsequently compared with one year historical outcomes data from 29 patients from a 2016 double-blind randomized controlled trial [8], where patients received intradiscal PRP therapy using a lower concentration of platelets (> 5 × PRP). Independent sample t-tests were used to assess differences in continuous variables, and chi-square tests were used to evaluate differences in discrete variables. A p-value < 0.05 was considered to be statistically significant.

## Results

### Patient flow

Eighty-one patients were retrospectively assessed for eligibility at a single outpatient interventional orthopaedic clinic. Forty-four patients did not meet inclusion criteria. The remaining 37 patients were included in the study (> 10 × PRP 2021 cohort).

### Demographics and follow-up intervals

The mean age was 42.7 ± 18.2 years (range: 14–72); 12 patients were between 14 and 29 years of age, 15 patients were between 30 and 55 years, and ten patients were between 50 and 72 years. There were 23 males and 14 females. The mean follow-up time from the date of procedure was 18.3 ± 13.3 months (range: 3–43 months; 3–6 months: n = 8; 7–12 months: n = 7; 13–24 months: n = 8; 25–43 months: n = 14). All but three patients reported a pain duration for seven months or more prior to the procedure, and 15 patients reported a pain duration for greater than 24 months.

### Clinical outcomes and patient satisfaction

**Fig. 1:**
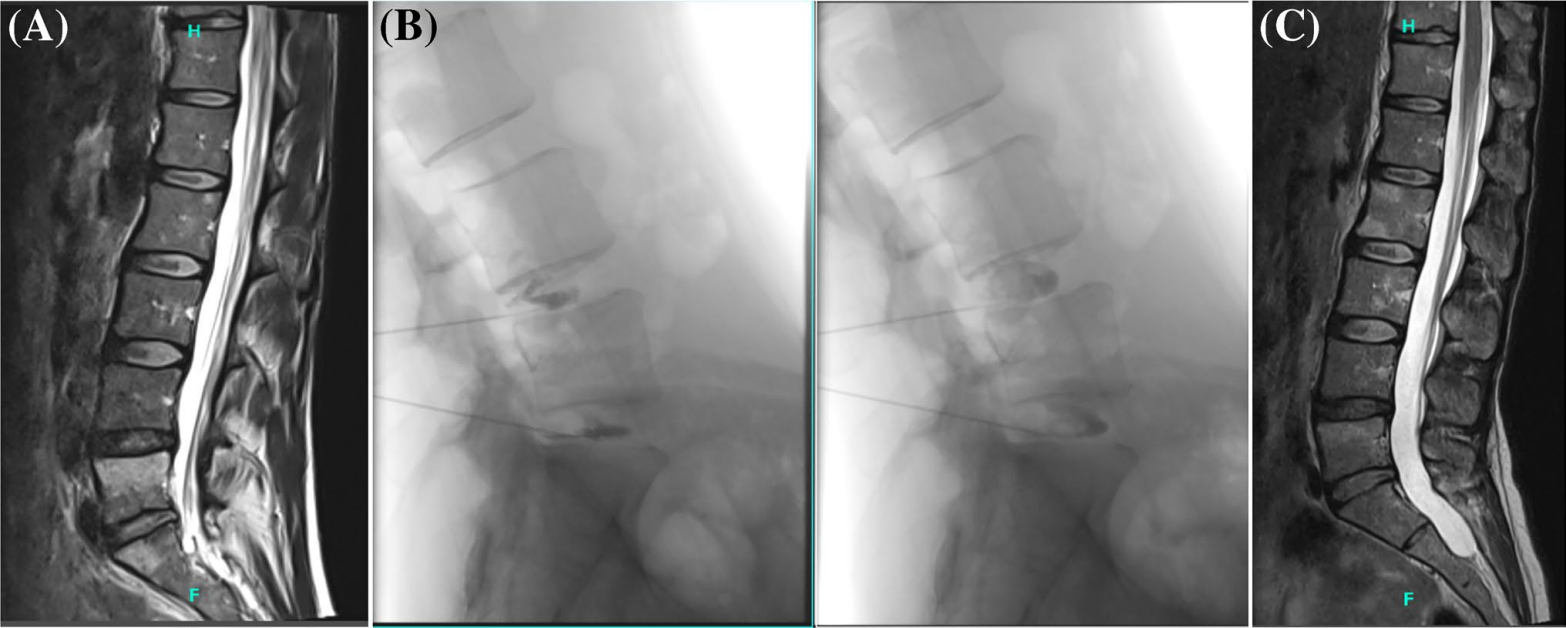
Representative magnetic resonance imaging (MRI). **A** MRI sagittal T2 image of Modic 1 changes at L4-5 and L5-S1 pre-treatment. **B** Lateral fluoroscopic images of intradiscal injection of contrast at L4-5 and L5-S1 showing annular fissures on the left and post-intradiscal leukocyte-rich platelet-rich plasma injections on the right. **C** MRI sagittal T2 image of improvement at L4-5 and L5-S1 at 1 year post-treatment

Pain and FRI scores are reported in Table 1. There were significant improvements in pain and FRI scores at the time of follow-up, with mean changes of 3.4 ± 2.5 and 46.4 ± 27.6% from baseline, respectively (p < 0.001 for both). MCIDs in pain and function were met by 73% of patients (n = 27) and 89% of patients (n = 33), respectively. Representative magnetic resonance imaging scans from a patient who demonstrated improvement following treatment are shown in Figs. 1A–1C.

**Table 1:** Pain and functional rating index scores

	> 10 × PRP 2021 Cohort (n = 37)		< 5 × PRP 2016 (Historical) Cohort (n = 29)		P-value (2021 vs. 2016)
	Mean (SD)	P-value (vs. baseline)	Mean (SD)	P-value (vs. baseline)	
Baseline NRS Pain Score	6.8 (2.0)		4.7 (2.2)		** < 0.001**
Follow-up NRS Pain Score	3.4 (2.4)	** < 0.001**	3.2 (2.4)	0.063	0.738
Change in NRS Pain from Baseline	3.4 (2.5)		1.7 (1.6)		**0.004**
Baseline FRI Score	65.3 (18.7)		51.5 (15.6)		**0.002**
Follow-up FRI Score	34.3 (21.8)	** < 0.001**	33.9 (20.4)	**0.001**	0.940
Change in FRI Score from Baseline	46.4 (27.6)		33.7 (12.3)		**0.016**

*FRI*, Functional Rating Index; *NRS*, numerical rating scale; *PRP*, platelet-rich plasma; *SD*, standard deviation

The majority of patients reported that they started noticing improvements in the first 12 weeks (n = 26; 70.3%) and were satisfied with their PRP injection (n = 30; 81%). Five (13.5%) patients noted that they were the same or worse than before the procedure and did not experience any improvements. The procedure was defined as successful if patients reported all of the following: (1) ≥ 2-point change in NRS pain score; (2) ≥ 9-point change in the FRI; and (3) satisfaction with the procedure. Twenty-six patients met the criteria, for a success rate of 70%. Seven patients (19%) did not meet any of the criteria for success and failed to improve at all. One patient had a complication of spondylodiscitis [18]. No patients experienced a progressive disc herniation post-procedure.

### Comparisons to outcomes in the historical cohort

For the historical (< 5 × PRP 2016) cohort, the mean change in pain score from baseline was 1.7 ± 1.6 (p = 0.063), and the mean change in FRI score from baseline was 33.7 ± 12.3 (p = 0.001). Compared to the historical cohort, the > 10 × PRP 2021 cohort started with worse pain and function scores and experienced significantly greater degrees of improvement in both pain and function (p = 0.004 and 0.016, respectively) (Table 1). In addition, the patient satisfaction rate was higher in the > 10 × PRP 2021 cohort compared to the historical cohort (81% vs. 56%; χ^2^ = 4.9; p = 0.027). Of note, age and gender distributions were similar between the > 10 × PRP 2021 cohort and the historical cohort (42.7 ± 18.2 vs. 41.4 ± 8.1 years; 38% vs. 52% females).

## Discussion

Emerging data have suggested that intradiscal PRP therapy with < fivefold increases in platelet concentrations may have therapeutic value for certain patients with CLDP [8, 13, 19, 20]. Our results add to the current literature showing long-term benefits of intradiscal PRP in the management of patients with CLDP [9, 14]. There were statistically and clinically significant improvements in patient-reported NRS pain and FRI scores at an average of 18 months following intradiscal injections of PRP with > tenfold increases in platelet concentration. Patient satisfaction and success rates were high. No other study has yet investigated clinical outcomes following intradiscal injections of > 10 × PRP.

Platelets have been shown to contain more than 1500 bioactive proteins, including growth factors, adhesive proteins, chemokines, and angiogenic factors [21]. In particular, platelet-derived growth factor (PDGF) and transforming growth factor β (TGF-β) are abundant in platelets and also play a role in healing [22]. Previous studies have demonstrated a positive linear relationship between platelet count and TGF-β or PDGF concentrations in PRP preparations [23]. As a correlation between higher platelet concentrations and improved patient-reported outcomes has been reported [11], it was hypothesized that PRP preparations containing higher platelet concentrations (> 10 ×) would further improve pain and functional outcomes in patients with CLBP, compared to PRP preparations containing lower platelet concentrations (< 5 ×). This was supported by our findings, which showed significantly greater degrees of improvement in pain and function in patients receiving intradiscal injections of > 10 × PRP than those receiving < 5 × PRP from a historical cohort [8]. This was observed despite the > 10 × PRP 2021 cohort having worse pain and function at baseline, compared to the historical cohort. In addition, patient satisfaction was higher in the current cohort, compared to the historical cohort (81% vs. 55%).

Although the success rate following intradiscal injections of > 10 × PRP was high in this cohort of CLDP patients, it is important to note that seven patients (19%) failed to improve at all. Future studies involving further characterization of the PRP injectate, including cell differentials and bioactive protein levels, and assessment of MRI criteria are needed to better elucidate why some patients do not experience any improvements following these injections.

There are several limitations to consider for this study. First, the follow-up time points were variable, due to the retrospective nature of the study. Second, baseline NRS pain and FRI scores in the current > 10 × PRP cohort were worse than those in the historical < 5 × PRP cohort; however, age and gender distributions were similar between cohorts. Lastly, shorter-term effects were not assessed in the study and should be investigated in the future.

Overall, there is a need for evidence-based, interventional treatments that address the underlying pathology of CLDP. Intradiscal PRP therapy has shown promising results in the CLDP literature, but there are wide variabilities in PRP type and processing. Results from this study suggest that clinical outcomes following intradiscal PRP injections in patients with CLDP can be optimized by using a PRP preparation with a higher concentration of platelets (> 10 ×). Future studies exploring PRP with higher concentrations of platelets in larger sample sizes and with longer follow-ups are warranted, as well as a more detailed analysis of the cellular composition of PRP (e.g., leukocyte-rich vs. leukocyte-poor) to assess other variables.

## Data Availability

The datasets generated during and/or analyzed during the current study are available from the corresponding author on reasonable request.

## References

[1] GBD 2017 Disease and Injury Incidence and Prevalence Collaborators (2018). Global, regional, and national incidence, prevalence, and years lived with disability for 354 diseases and injuries for 195 countries and territories, 1990–2017: a systematic analysis for the Global Burden of Disease Study 2017. Lancet.

[2] Wu A, March L, Zheng X, Huang J, Wang X, Zhao J, Blyth FM, Smith E, Buchbinder R, Hoy D (2020). Global low back pain prevalence and years lived with disability from 1990 to 2017: estimates from the Global Burden of Disease Study 2017. Ann Transl Med.

[3] United States Bone and Joint Initiative (2014) The Burden of Musculoskeletal Diseases in the United States (BMUS), Third Edition. Rosemont, IL

[4] Schwarzer AC, Aprill CN, Derby R, Fortin J, Kine G, Bogduk N (1995). The prevalence and clinical features of internal disc disruption in patients with chronic low back pain. Spine (Phila Pa 1976).

[5] DePalma MJ, Ketchum JM, Saullo T (2011). What is the source of chronic low back pain and does age play a role?. Pain Med.

[6] Verrills P, Nowesenitz G, Barnard A (2015). Prevalence and characteristics of discogenic pain in tertiary practice: 223 consecutive cases utilizing lumbar discography. Pain Med.

[7] Peng B-G (2013). Pathophysiology, diagnosis, and treatment of discogenic low back pain. World J Orthop.

[8] Tuakli-Wosornu YA, Terry A, Boachie-Adjei K, Harrison JR, Gribbin CK, LaSalle EE, Nguyen JT, Solomon JL, Lutz GE (2016) Lumbar Intradiskal platelet-rich plasma (PRP) injections: a prospective, double-blind, randomized controlled study. Pm r 8:1–10; quiz 10. 10.1016/j.pmrj.2015.08.010 10.1016/j.pmrj.2015.08.010 26314234

[9] Monfett M, Harrison J, Boachie-Adjei K, Lutz G (2016). Intradiscal platelet-rich plasma (PRP) injections for discogenic low back pain: an update. Int Orthop.

[10] Marx RE, Harrell DB (2014). Translational research: the CD34+ cell is crucial for large-volume bone regeneration from the milieu of bone marrow progenitor cells in craniomandibular reconstruction. Int J Oral Maxillofac Implants.

[11] Jain D, Goyal T, Verma N, Paswan AK, Dubey RK (2020). Intradiscal platelet-rich plasma injection for discogenic low back pain and correlation with platelet concentration: a prospective clinical trial. Pain Med.

[12] Akeda K, Ohishi K, Masuda K, Bae WC, Takegami N, Yamada J, Nakamura T, Sakakibara T, Kasai Y, Sudo A (2017). Intradiscal injection of autologous platelet-rich plasma releasate to treat discogenic low back pain: a preliminary clinical trial. Asian Spine J.

[13] Levi D, Horn S, Tyszko S, Levin J, Hecht-Leavitt C, Walko E (2016). Intradiscal platelet-rich plasma injection for chronic discogenic low back pain: preliminary results from a prospective trial. Pain Med.

[14] Cheng J, Santiago KA, Nguyen JT, Solomon JL, Lutz GE (2019). Treatment of symptomatic degenerative intervertebral discs with autologous platelet-rich plasma: follow-up at 5–9 years. Regen Med.

[15] Prysak MH, Kyriakides CP, Zukofsky TA, Reutter SE, Cheng J, Lutz GE (2021). A retrospective analysis of a commercially available platelet-rich plasma kit during clinical use. Pm r.

[16] Childs JD, Piva SR, Fritz JM (2005). Responsiveness of the numeric pain rating scale in patients with low back pain. Spine (Phila Pa 1976).

[17] Copay AG, Glassman SD, Subach BR, Berven S, Schuler TC, Carreon LY (2008). Minimum clinically important difference in lumbar spine surgery patients: a choice of methods using the Oswestry Disability Index, Medical Outcomes Study questionnaire Short Form 36, and pain scales. Spine J.

[18] Beatty NR, Lutz C, Boachie-Adjei K, Leynes TA, Lutz C, Lutz G (2019). Spondylodiscitis due to Cutibacterium acnes following lumbosacral intradiscal biologic therapy: a case report. Regen Med.

[19] Chang MC, Park D (2021). The effect of intradiscal platelet-rich plasma injection for management of discogenic lower back pain: a meta-analysis. J Pain Res.

[20] Muthu S, Jeyaraman M, Chellamuthu G, Jeyaraman N, Jain R, Khanna M (2021) Does the intradiscal injection of platelet rich plasma have any beneficial role in the management of lumbar disc disease? Global Spine J:2192568221998367. 10.1177/2192568221998367 10.1177/2192568221998367 PMC9121148 33840260

[21] Boswell SG, Cole BJ, Sundman EA, Karas V, Fortier LA (2012). Platelet-rich plasma: a milieu of bioactive factors. Arthroscopy.

[22] Anitua E, Andia I, Ardanza B, Nurden P, Nurden AT (2004). Autologous platelets as a source of proteins for healing and tissue regeneration. Thromb Haemost.

[23] Sundman EA, Cole BJ, Fortier LA (2011). Growth factor and catabolic cytokine concentrations are influenced by the cellular composition of platelet-rich plasma. Am J Sports Med.

